# Local knowledge in community-based approaches to medicinal plant conservation: lessons from India

**DOI:** 10.1186/1746-4269-2-20

**Published:** 2006-04-07

**Authors:** Shailesh Shukla, James Gardner

**Affiliations:** 1 Natural Resources Institute, University of Manitoba, Winnipeg, MB, R3T2N2, Canada

## Abstract

**Background:**

Community-based approaches to conservation of natural resources, in particular medicinal plants, have attracted attention of governments, non governmental organizations and international funding agencies. This paper highlights the community-based approaches used by an Indian NGO, the Rural Communes Medicinal Plant Conservation Centre (RCMPCC). The RCMPCC recognized and legitimized the role of local medicinal knowledge along with other knowledge systems to a wider audience, i.e. higher levels of government.

**Methods:**

Besides a review of relevant literature, the research used a variety of qualitative techniques, such as semi-structured, in-depth interviews and participant observations in one of the project sites of RCMPCC.

**Results:**

The review of local medicinal plant knowledge systems reveals that even though medicinal plants and associated knowledge systems (particularly local knowledge) are gaining wider recognition at the global level, the efforts to recognize and promote the un-codified folk systems of medicinal knowledge are still inadequate. In country like India, such neglect is evident through the lack of legal recognition and supporting policies. On the other hand, community-based approaches like local healers' workshops or village biologist programs implemented by RCMPCC are useful in combining both local (folk and codified) and formal systems of medicine.

**Conclusion:**

Despite the high reliance on the local medicinal knowledge systems for health needs in India, the formal policies and national support structures are inadequate for traditional systems of medicine and almost absent for folk medicine. On the other hand, NGOs like the RCMPCC have demonstrated that community-based and local approaches such as local healer's workshops and village biologist program can synergistically forge linkages between local knowledge with the formal sciences (in this case botany and ecology) and generate positive impacts at various levels.

## Background

### Purpose and objectives of the research

The contribution of local knowledge systems in conservation and sustainable use of natural resources is gaining wider recognition [1]. Local knowledge associated with the use and conservation of medicinal plants is either codified in ancient scriptures or is folk-based and transmitted through generations in the form of community-based health traditions. The codified knowledge has managed to expand globally through official recognition and some corresponding policy and financial support. The largely undocumented folk-based knowledge and traditions, on the other hand, have survived on their own in the absence of any official recognition and policy and administrative support by governments at the state and national levels [2]. The problems, progress, and prospects of folk and codified streams are therefore distinct, with the former deserving more attention. Despite the lack of official recognition and support, the recent efforts and approaches of grassroots community groups and NGOs in revitalizing and promoting folk knowledge systems of medicine are noteworthy. The purpose of this paper is to describe and analyze such an NGO-led approach from India in order to demonstrate the contributions of local knowledge systems in generating positive outcomes at the local, the regional, the national and the global levels. The specific objectives of the paper are:

i) to describe the community-based approaches taken by the RCMPCC (Rural Commune's Medicinal Plant Conservation Centre) that successfully used folk and codified local medicine systems along with formal medical systems

ii) to understand the outcomes of these community-based approaches at the various levels in order to derive lessons for policy and practice in regard to community-based medicinal plant conservation

### The global status of local medicinal plant knowledge systems

Medicinal plants are gaining wider recognition in recent initiatives for conservation and development at the global level. This is evident in the vision and mission statement of World Health Organization (WHO) on health improvement and in community-based conservation initiatives by international organizations, including the World Bank, the International Development Research Centre (IDRC) and UNDP, for example. The effort by the WHO to recognize and promote the use of local medicinal plant knowledge systems in the health sector, particularly in developing countries, is prominent. The terminologies related to a use of plant-based medicine vary in different cultures, countries, and communities. The WHO, in its widely acknowledged report, uses an umbrella term 'traditional medicine' to describe such uses and offers its working definition as 'diverse health practices, approaches, knowledge and beliefs incorporating plant, animal, and/or mineral based medicines, spiritual therapies, manual techniques and exercises applied singularly or in combination to maintain well-being, as well as to treat, diagnose or prevent illness' [6]. The scope of this paper is limited to practices/approaches/knowledge with respect to only plant-based medicines.

The World Bank report [7] indicates that more than eighty percent of the population of South Asia uses plant-based medicines for maintaining and improving their health. The total reported usages of medicinal plants vary. For instance, the WHO [6] study lists 21,000 plants with reported medicinal uses around the world, while Schippmann and co-workers [8] estimated this figure as 52,885. Amidst these conflicting claims on numbers, the use of medicinal plants by local communities or groups has remained high. Such local uses of medicine, are again known by different terms but can be classified broadly into the following three categories[9]: (i) Traditional Systems of Medicine (TSM), with a systematic codified body of knowledge either in the form of pharmacopoeias or ancient scriptures [Ayurvedic, Chinese and Tibetian medicine, Siddha, Unani (Arabic) etc.]; (ii) Traditional Medical Knowledge or Folk Medicine, which is transmitted by oral means and is mostly acquired through learning-by-doing approaches, and (iii) Shamanistic or Spiritual Medicine, with a strong religious/spiritual element and can be practiced only by highly specialized local experts called 'shamans'. In general, except for TSM, the legal and political recognition and support for folk and spiritual medicine at the national and the global level (see, Table 1) has remained weak [10,11]. The lack of official and governmental support, compounded by the devaluation of the folk knowledge [12] by local communities and societal systems at large, has resulted in the erosion of the local medicinal plant knowledge systems. The issues related to the formal recognition of TSM, and the lack thereof for other medicinal plant knowledge systems (folk and spiritual), have some things in common. However, in most cases, the distinct lack of attention to the latter systems suggests that they deserve further consideration in countries like India, China and South Africa, where a reliance on TSM and folk medicine is more widespread and evident.

**Table 1:** Comparisons of the features and policy issues of local medicinal plant knowledge: The folk and codified systems of medicine in India

*Folk traditions of local medicinal plant knowledge*	*TSM of local medicinal plant knowledge*
Originated in communities to meet daily healthcare/survival needs, largely undocumented	Originated by scholars, physicians and seers and documented in manuscripts/Vedic texts(1000–1500 BC), scriptures for human well-being and developed as a classified main branches
Transmission multigenerational and by oral means through learning-by doing and through more than 300 formal educational colleges	Transmission is often institutionalized through written texts and hands-on training
Mainly empirical, adapted	Sophisticated philosophical and theoretical roots with a scope for refinement
No legal status, No budgetary allocation, on the contrary vulnerable to disregard and devaluation	Legal status as 'Indian Systems of Medicine' with five percent of budgetary allocation (health) wider social and official acceptance and recognition
Approximate # practitioners are 600,000 birth attendants, 60,000 bone setters, 100,000 herbal healers, 60,000 healers specialized in treating poisonous snake bites and millions of households/women	Approximately 600,000 registered medicinal practitioners, out of which, 10 percent practice medicine on the basis of TSM.
Uses more than 7,500 medicinal plants	The four streams of Ayurvedic, Unani, Siddha and Tibetan uses approximately 4,500 medicinal plants
**POLICY LEVEL ISSUES**	
Local state and national incentives for systematic documentation and dissemination needed	Available documentation in Sanskrit at scattered places, interpretation and consolidation in a commonly-understood language will facilitate further use/research
In-depth understanding of and incentives for (local/state/national/global) incentives can facilitate transmission	Formal institutions for transmission are present but are poorly funded
Sustaining interest and apprenticeship of the younger generations is a challenge	Maintaining quality and standards of practitioners is a challenge
Scope of learning from TSM and allopathic medicine system is limited due to access, affordability and literacy issues at the community level	Both TSM and allopathic medicine draw heavily on the folk system for herbal remedies or drugs without giving credit or sharing benefits to local communities
Benefit sharing mechanisms are developing and difficult to implement at community level	Well-established and implemented benefit sharing mechanism in the form of patent/trademarks and other forms of protection
Efficacy, standardization and safety studies using scientific parameters are almost nil due to lack of authentic documentation and neglect by official policies	Efficacy, standardization and safety studies are not encouraged due to high-cost (200,000 US$) and time consuming (8–10 years) scientific validation and language barriers
Collaboration by other stakeholders is difficult and confined to documentation/dissemination efforts	Collaboration is generally encouraged if the epistemological and philosophical foundations are matching

Sources: Compiled based on Shankar (2001)[11], Shankar and Venkatasubramanian (2004)[15] and WHO (2002)[6]

### The local medicinal plant knowledge systems in India

In a country like India, where 65 percent of the total population has access to only local medicinal plant knowledge systems [6], and 70 percent of the population lives in villages struggling to access and afford modern allopathic medicines, both TSM and the folk knowledge systems of medicine are of significance[13] TSM exists in the form of well-known classical traditions of Ayruevda, Unani and Siddha, which are characterized by a large number of practitioners trained through formal institutions, a well-codified body of texts either in the form of the scriptures or other written forms and an official recognition as 'Indian Systems of Medicine'[14]. Both Ayurvedic and Siddha systems of medicine originated more than 3000 years ago and were prevalent in North and South India, respectively. The Unani system of medicine originated in Greece (460–377 BC), and became more popular in India after the establishment of the Central Council for the Research in Indian Medicine and Homeopathy in 1969. Complementary to these codified systems, the folk knowledge systems are largely transmitted through oral means and flourish at the village level [15] with little or negligible support from official channels at the state or the national level. The features and policy issues concerning these systems are compared in Table 1.

Although folk knowledge ranks at the top in terms of the total number of users, the number of medicinal plants used and number of practitioners, the desired policy support for its recognition and development is not evident. There is some policy and legal support for the codified systems of Ayurvedic, Unani and Siddha in India [6] but it is inadequate [15].

International organizations like UNDP, IDRC, OXFAM, WHO, Ford Foundation and the World Bank are the leading sources of funding for broad-based programs dealing with medicinal plant conservation and development in India and Asia [16]. The funding and capacity building support of these agencies to Indian governments and NGOs is predominant in developing state, national, and regional visions and strategies for participatory, comprehensive, and sustainable management of medicinal plants. At a national level, the Department of the Indian Systems of Medicine (Health) and the Medicinal Plant Boards [17] are the most relevant government agencies that deal directly with the medicinal plant sector and associated local knowledge systems. These national level efforts are more recent and more targeted at improvement in codified streams such as TSM. Further, their direction, design and delivery have largely been limited to the state level. At the sub-state level (district, block or taluka and village), the programmatic interventions related to medicinal plant and associated local knowledge systems are organized by local NGOs and community groups.

The most notable and oldest among these NGOs is the Foundation for Revitalization of Local Health Traditions (FRLHT) [2], which has been identified and supported by the Government of India as a 'centre of excellence' in the field of medicinal plants. FRLHT's programs and activities in this sector are largely confined to peninsular and Southern India. Nevertheless, FRLHT has inspired similar initiatives in other parts of India and Asia through networking and capacity building for other NGOs. The RCMPCC, based in the western state of Maharashtra is one such NGO. The RCMPCC has demonstrated that the use of community-based participatory approaches at the local level facilitates learning among various stakeholders and provides a platform for interactions among the formal botanical knowledge, TSM and the folk knowledge systems relevant to medicinal plants at the state level. The learning resulting from these local knowledge-based approaches has transformed the agenda of medicinal plant conservation at the sub-state or state level in Maharashtra and has the potential to enrich and inform stakeholders at the national and global levels.

## Methods

Before the start of field research activities, a series of consultations was held at the Center for Community-based Natural Resources Management at Natural Resources Institute (NRI), University of Manitoba, Winnipeg, Canada between April and October 2003 as a part of the Equator Initiative of the United Nations Development Program (UNDP). This helped in the selection of communities, research objectives and field research methods. The RCMPCC of Pune, India was one of the four Equator Initiative sites selected as part of the study. The purpose, objectives and research design were based on an interactive adaptive approach as suggested by Nelson [3] and were shared with key leaders and field staff of RCMPCC. The RCMPCC had established thirteen project sites in the rural, tribal and forested areas known as medicinal plan conservation areas across the state of Maharashtra. One of their project sites near the village of Amboli was chosen as a case study, on the basis of the following criteria:

***a) Willingness of the community/project functionaries to participate: ***The willingness and oral consent of villagers were addressed and obtained in local dialects during an initial workshop in Amboli that was attended by the village panchayat, i.e., local management committee members. Special efforts (e.g. personal visits or informal conversations while walking in forest) to ensure written consent and active participation of special groups, such as women and local healers, were made.

***b) Evidence of use of local knowledge in sustainable management of local biodiversity: ***Amboli has well-documented evidence for community-based conservation and is rated as one of the best examples of such (267.63 hectares) by the RCMPCC [4].

A review of literature was undertaken on issues related to medicinal plants and associated local knowledge systems to contextualize the problem as described in the background. The field research used a variety of qualitative research tools [5] such as semi-structured interviews with key people (N = 12) from the RCMPCC, local healers (N = 11, seven male and four female), community members (N = 5) and Forest Department officials (N = 3). The selection of the research participants was limited to those who were involved in designing, implementing and/or participating in the two community-based approaches (i.e. *vaidu sammelan *and village biologist program) of RCMPCC. For the purpose of this paper, data relevant to the outcomes (as perceived as 'benefits' or 'visible impact' by the research participants) were collected and analyzed. The senior author stayed in Amboli for more than three months and participated in a village botanist training program organized at Amboli (December 2003) and *vaidu sammelan *(March 2004). The field research was complemented by a review of relevant documents and internal documents (such as proceedings of the village botanists' workshops (December 2000 and November 2001) provided by RCMPCC.

The Joint-Faculty Research Ethics Board at University of Manitoba approved the research protocols (Protocol No. J2003:141) for this study.

## Results and discussion

This section describes and analyzes two community-based approaches undertaken by RCMPCC, which combine both local (folk and codified) and formal systems of medicine to demonstrate that such creative integration at the local level can generate positive impacts at all levels.

### Building on medicinal plant knowledge of local healers: the RCMPCC way

The RCMPCC initiative was envisioned by a Mumbai-based NGO, called Rural Commune for the In-Situ Conservation and Sustainable Utilization of the Medicinal Plant Diversity of Maharashtra, through developing partnerships among the Forest Department, local communities and NGOs. To this end, the RCMPCC, in collaboration with other stakeholders, organized several activities, such as the establishment of a network of 13 Medicinal Plant Conservation Area or MPCAs (each ranging from 250–400 hectares) in Maharashtra. The MPCAs were selected in consultation with the Forest Department, local communities, and available scientific literature, based on the following criteria:

• Relatively undisturbed forest areas representing different bio-climatic zones

• Forest areas with rich biodiversity

• Areas with natural availability of water

• Locally and otherwise known for harboring medicinal plants.

The MPCAs were legally notified by the Maharashtra Forest Department as permanent medicinal plant reserves and this inspired other state governments and the Government of India to include them in their conservation and development agenda. The RCMPCC also completed the documentation of some 150 species in the Medicinal Plant Conservation Areas through participatory approaches like the village biologist program, the Conservation Assessment and Management Program, local healers' conventions and scientific assessments by field botanists. They created village management structures such as Local Management Committees (LMCs) and Self-Help Groups (SHGs) for marketing and local sale of herbal products [18].

**Figure 1:**
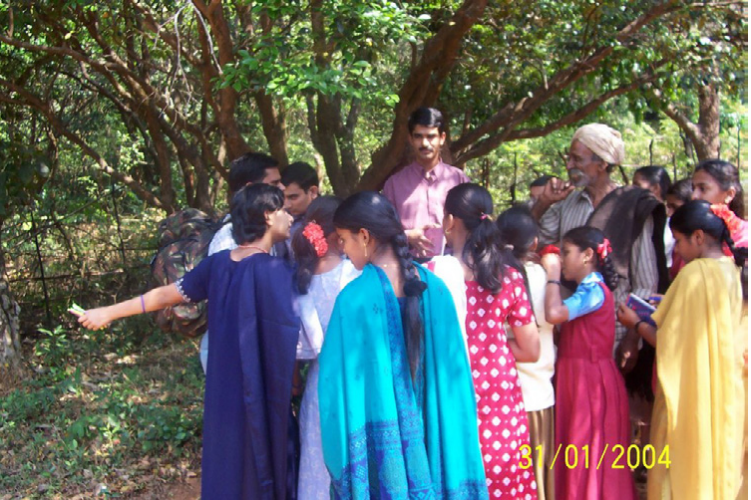
Local *vaidus*, RCMPCC scientists and school students engaged in knowledge exchange during the village biologist workshop in Amboli (Photo- JPEG format)

A group of individuals known as local knowledge experts or healers and ecological experts called *vaidus *were identified by the RCMPCC. The term *vaidus *is a generic folk term most commonly used in the Marathi and Hindi languages to describe healers and traditional herbal practitioners, used most commonly for, but not limited to, male healers. These *vaidus *possessed extraordinary knowledge and interest in the local plants, fauna and ecosystems. Many of the *vaidus *practiced herbal treatments for diseased humans, livestock, and crops and developed skills in identification and use of locally grown plants. The RCMPCC recognized the creative potential of the *vaidus *as village biologists (VB) or barefoot botanists in the conservation and management of medicinal plant diversity through sustainable uses [19]. To build effective partnerships with the local knowledge experts, the RCMPCC organized two programs: 1) *vaidu sammelan *or local healer's workshops, and 2) the village biologists (previously known as barefoot botanists) training programs. FRLHT initiated the Barefoot Botanist (BFB) program in 1995 with the original aim to enrich the local medicinal plant knowledge of the village *vaidus *with the relevant formal botanical skills. The trained *vaidus *were expected to perform better in their own profession and in providing guidance to eco-tourists and researchers. RCMPCC prefers the use of more inclusive term 'village biologist' instead of 'barefoot botanist'. These programs also helped in mutual learning and the strengthening of the capabilities of formally-trained botanists and local *vaidus *through dialogue and exchange of knowledge. (See, Figure 1)

The purpose of *vaidu sammelan *was to: 1) document the knowledge of local *vaidus *about plants; 2) encourage value-added activities by promoting local use and sale of herbal products; 3) provide a platform for *vaidus *to demonstrate their products; and, 4) to provide a platform for the *vaidus *and other health practitioners to interact and encourage participation in local biodiversity conservation efforts. The village biologist program was broad in terms of coverage, with 3 or 4 local experts identified at each location. Selection of village biologists was based on: 1) good knowledge of local plants and their uses and cultural significance; 2) interest in local environmental and conservation issues; and, 3) ability to read and write. Most village biologists identified were *vaidus*, forest guards and knowledgeable elders.

### Learning from the outcomes of RCMPCC's community-based approaches

The discussion in this section draws mainly on interviews with selected informants (as indicated in methods) and a review of relevant documents.

Table 2 highlights some of the major outcomes of these two methods, along with the policy implications, at various levels of activity. These levels are geographical units or operations at which the various activities of the RCMPCC has generated visible impacts, as perceived by RCMPCC and the communities including the *vaidus*.

**Table 2:** Major outcomes of RCMPCC's community-based programs

**Major Outcome**	**Levels of influence**				
***Policy implication***	**Village/Sub-district**	**District (MPCA)**	**State (RCMPCC)**	**National**	**Global**
Documentation of traditional knowledge of medicinal plants from *vaidus- *both men and women *Systematic documentation created opportunities for collaborative research with TSM*	Role of women *vaidus *recognized Involvement of *vaidus*/VB in village development/conservation activities	Two local language booklets on ethno botanical information about plants and *vaidus*	Systematic data base on 326 medicinal plants, 465 herbal formulations, Illness-specific database on 265 plants, herbarium record of 804 species	Raw drugs formulations of 75 plants available for further scrutiny	VB, Vaidus training institutionalized in GEF programs in nine states
Local assessment of Rare, Endangered and Threatened (RET) species *Potential of Local medicinal knowledge alongside TSM as inputs in official planning*	Local monitoring by panchayats (village councils), LMCs, SHGs. RET List maintained at two MPCA by local communities with the support of VBs/*vaidus*	Training of 36 selected VB and district forest officers on RET monitoring Incorporating list in district working plans of Forest Department	Prioritized species identified and raised in seven forest nurseries of the state Helped FD in species recovery and enrichment programs	Unique species highlighted	Unique specie of Global importance identified at Leghapani MPCA
Recognition and use of *vaidus* *Official recognition of Local knowledge experts (restricted up to sub-state level)*	*Vaidus *Involved in selection of appropriate seedlings in forest nurseries *Vaidus *and VB got 'official' recognition to practice their knowledge	Employing *vaidus *as field guides/eco-tourism Honouring VB/*vaidus *in two workshops	State level database of *vaidus *and village botanists according to their area of expertise (RCMPCC) CAMP exercises	Display and dialogue with other national level *vaidus *through two national herbal expos	Local *vaidus *guided ITTO (Japan) research scientists as research anchors in vegetation mapping
Opportunities for collaborative research *Collaborative Research mainly initiated at national/below level and often self-inspired/funded*	A herbarium preparation techniques learned by VB and *vaidus *through botanists Validation of *vaidus*/VB's local knowledge through sharing/exchange with botanists	Selected VB/*vaidus *as trainers for other MPCA selected Study in sacred groves by RCMPCC	Market study of 22 medicinal plants Biodiversity assessment of select MPCAs RCMPCC as a resources institution in State medicinal Plant Board	Pilot project on standardization/cultivation processing of prioritized species in six MPCAs supported by Department of Science and Technology	
Transmission of folk knowledge facilitated *Systematic efforts for understanding transmission are lacking at State/National/Global levels*	People Biodiversity register at one village Herbarium sheets demonstration in three schools	11 demo gardens, 10 interpretation center highlighting contributions of VB/*vaidus*,40 home herbal gardens		Ministry of tribal affairs supported study on people's biodiversity register	

Source: self-compiled

At the village level, the local medicinal plant knowledge of the *vaidus *is widely recognized as 'valid' alternative systems of knowledge. 'This is the most commonly available knowledge in our village and people often seek our help, even during odd times such as night. We treat many villagers who have snake bites during night or are injured by black bear when we work in our farms near forest' (SA, local vaidu, Amboli, March, 2004). Amboli has a Public health centre but the continuous availability of allopathic practitioners is normally not the case. The accessibility of *vaidus *at anytime, therefore, is regarded as one of the major benefits for the local communities. 'Approaches such as the *vaidu *sammelan give our *vaidus *a community-recognized local licence to practice' (MG, Community leader, Amboli, January, 2004). In addition, the village biologist program provides avenues to facilitate the use of the local medicinal plant knowledge of the *vaidus *by the local formal institutions in two ways: i) *vaidus' *knowledge about the rare and endemic medicinal plants was used in the selection of plant species in the nurseries by the Forest Department ; ii) *vaidus *learned the herbarium preparation techniques from the botanists during the village biologist program and applied their training by conducting demonstrations of local plants through these herbarium sheets in secondary schools. The People's Biodiversity Register program has been initiated at Chavni village in the Amba Valley MPCA through consultation with the *vaidus *and villagers. This 100 page register was prepared in English and Marathi language. It contains useful information about the local biodiversity in and around Chavni village and details about the *vaidus*. The register has been useful in local biodiversity assessment, conservation and monitoring.

The *vaidus *of all thirteen Medicinal Plan Conservation Areas were involved in the identification of Rare, Endangered and Threatened (RET) plant species. At the Amboli and Leghapani MPCAs, *vaidus *generated a list (in the local language) and photographs of these species, for regular monitoring and regeneration in villagers' home gardens. 'We share the photographs and lists of rare plants with tourists and researchers, so they become sensitive to these plants when they move around in forest' (KG, local *vaidu*, Amboli, March 2004).

Participation of women *vaidus *in these programs has helped in their improved recognition. 'In the past, our expertise was perceived to midwifery skills. But now we also treat people as male *vaidus *do. Besides, our participation in programs like *vaidu sammelan *gives us a confidence and authority to take part in village conservation and development activities related to medicinal plans along with male *vaidus*' (JS, women *vaidu*, Amboli, March 2004). The women *vaidus *in particular and *vaidus *in general, are being regarded as equal partners, not only in generating a useful knowledge base about medicinal plants but also in developing an agenda for their conservation and sustainable use.

At the district level, the Forest Department publicized the contributions of the *vaidus *through a special publication or in their working plans. For example, the district Forest Department of Sindhudurg and RCMPCC published a Marathi language booklet with the list and uses of 100 local medicinal plants. The Latin, English and vernacular names of the plants are listed along with their family names. In addition, a list of 113 *vaidus *with their areas of specialization and addresses is provided in the book. The book has been widely circulated among state forest officials, other state government departments, universities and NGOs. 'We have publicly honoured *vaidus *from Amboli. Some *vaidus *that were listed in the booklet are now being invited by NGOs and Other government departments to participate in training and share their stories' (RN, Forester, Sidhudurg Division, December 2003). The Forest Department has also recognized *vaidus *as eco-guides in Koynanagar. RCMPCC complemented these efforts by involving *vaidus *in developing and maintaining demonstration gardens, home herbal gardens and interpretation centres and in documenting sacred groves (the local areas that were conserved by villagers for religious or spiritual purposes).

At the state level, the collective documentation and collaborative research with the *vaidus*, helped in creating a database of medicinal plants and herbal formulations. These databases provided useful information about the Rare, Endangered and Threatened (RET) plant species that were prioritized for conservation and regeneration in the working plans of Forest Department. 'The research, documentation and database development efforts of RCMPCC with *vaidus *and communities, earned us an honour of 'Resource institution' in the State Medicinal plant board and Forest Department' (RCMPCC management board representative, Pune, March 2004).

At the national level, these approaches mobilized funding support for the two studies from the Department of Science and Technology and the Ministry of Tribal affairs on standardization of selected medicinal plants in six MPCAs and a biodiversity register in one MPCA, respectively. *Vaidus *from all thirteen MPCAs (three from each site) participated in the National Herbal Expo in 2000 and 2001 where they had shown their herbal products and had an opportunity to interact with government officials, NGO representatives and *vaidus *from other states.

The most visible national and global impacts of these approaches were the inclusion and recognition of *vaidus *as important project stakeholders in the new conservation and development funding plans that nine other states of India have submitted to the Global Environment Facility (GEF) for funding. The unique endemic plant species of *Ceropegia *spp. was discovered in Leghapani MPCA. [20]. 'Our *vaidus *have guided researchers form International Tropical Timber Organisation (ITTO) from Japan and helped them organize transects by identifying suitable pockets' (MA, Key leader, RCMPCC, December, 2003).

These impacts and outcomes constitute, what scholars of community-based conservation describe as 'benefits'- equity and empowerment being the key considerations [21]. The *vaidus *and their medicinal plant knowledge, have been recognized, legitimized and in some cases built upon to meet the medicinal plant conservation and development goals at various levels.

It is evident from the material in Table 2, that the activities of the RCMPCC generated some useful outcomes at the village, regional, state and national levels, rather than the global level. The national level outcomes (largely driven by the RCMPCC) include: the mobilization of funding commitments through pilot research projects on RET species, a biodiversity register in one MPCA and the recognition of the contributions of the *vaidus *in country-wide publications and national exhibitions. These outcomes have yielded some legitimization to and official use of the local medicinal plant knowledge and its holders (*vaidus*). At the state level, the recognition and support to the *vaidus *reflected through examples of the documentation on botanical and local nomenclature for plants, validation of medicinal uses of plant (as reported by the (*vaidus*) by Ayurvedic practitioners and botanists and collaboration with formal scientists for collective identification of the priority areas/species for conservation, simple resource mapping and herbarium development. At the global level, there has been some acknowledgment of the contributions of *the vaidus *as evident from their involvement in scientific studies.

The availability and acceptability of *vaidus *at the village level and their legitimacy, recognition and partnerships at the state and national levels, created a sense of empowerment (that they can decide and implement local conservation and development agenda) and equity (that their knowledge can inform and interact with knowledge of formally trained botanists or foresters). While these most sought-after benefits of community-based conservation may be achieved at the village and district levels, their fullest potential is yet to be realized at the state, national and global level.

## Conclusion

In India, although the majority of the population still relies on local knowledge systems to meet their health needs, the official policies and national support structures are inadequate for TSM and almost absent for folk medicine. NGOs like the RCMPCC have demonstrated that community-based approaches such as the *vaidu *sammelan and the village biologist programs can provide a platform on which holders of local medicinal plant knowledge systems (both folk and TSM) can interact with the holders of formal knowledge (e.g. botanists and other scientists). These approaches have also generated positive outcomes at different levels, such as the legitimization and recognition of the folk knowledge of the village *vaidus *in the district, state and (externally-aided) national plans of the Forest Department, the mobilization of collaborative research and funding commitments by the government departments, NGOs and research institutions at the state and the national levels and, most importantly, the pre-testing of community-based educational models for facilitating transmission of folk knowledge associated with uses of medicinal plants at the village or the sub-state levels. These outcomes show a way to achieve the larger goals of equity and empowerment as conceived in community-based conservation. In this case of community-based medicinal plant conservation, however, the achievement of these important goals is limited to local, district and, to a certain extent, state level. In order to make these goals more durable and widespread, such community-based approaches that build on local medicinal plant knowledge systems need to be encouraged with supportive policy and legislative measures at the national and the global levels.

## Competing interests

We do not have any monetary competing interest. This research was undertaken as a part of PhD thesis and therefore authors have academic interest.

## Authors' contributions

SS designed, carried out and analyzed the field research. JG helped in conceptualization of the study, supervised the study and significantly contributed in the improvement of this manuscript. All authors read and approved the final manuscript.
